# Quercetin Intake and Absolute Telomere Length in Patients with Type 2 Diabetes Mellitus: Novel Findings from a Randomized Controlled Before-and-After Study

**DOI:** 10.3390/ph17091136

**Published:** 2024-08-29

**Authors:** Aikaterini E. Mantadaki, Stella Baliou, Manolis Linardakis, Elena Vakonaki, Manolis N. Tzatzarakis, Aristides Tsatsakis, Emmanouil K. Symvoulakis

**Affiliations:** 1 Clinic of Social and Family Medicine, Department of Social Medicine, School of Medicine, University of Crete, 70013 Heraklion, Greece; linman@med.uoc.gr (M.L.); esymvoulakis@uoc.gr (E.K.S.); 2 Laboratory of Toxicology, Medical School, University of Crete, 71003 Heraklion, Greece; s.baliou@med.uoc.grevakonaki@med.uoc.grtzatzarakis@med.uoc.gr (M.N.T.); tsatsaka@uoc.gr (A.T.)

**Keywords:** type 2 diabetes mellitus, nutraceutical, quercetin, randomized trial, nutri-senotherapy, hypoglycemic effect, phytotherapy, integrated care, senolytic, telomere length

## Abstract

Telomeres, the protective chromosomal ends, progressively shorten and potentially are implicated in the pathogenesis of age-related diseases. In type 2 diabetes (T2DM), telomere shortening may play an important role, but the whole ‘picture’ remains limited. From a therapeutic perspective, the phytonutrient quercetin appears to be clinically effective and safe for patients with T2DM. Considering the above, we aimed to examine whether quercetin could interfere with telomere length (TL) dynamics. One hundred patients with T2DM on non-insulin medications registered within a primary healthcare facility were stratified by age and sex and randomly assigned to either standard care or standard care plus quercetin (500 mg/day) for 12 weeks, succeeded by an 8-week washout period and another 12 weeks of supplementation. Of the 88 patients completing the trial, 82 consented to blood sampling for TL measurements. Health assessments and whole blood absolute TL measurements using quantitative polymerase chain reaction (qPCR) were conducted at baseline and study end, and the findings of this subcohort are presented. Quercetin supplementation was associated with a significant increase in mean TL (odds ratio ≥ 2.44; *p* < 0.05) with a strengthened association after full adjustment for potential confounders through multiple logistic regression analysis (odds ratio = 3.48; *p* = 0.026), suggesting it as a potentially promising supplementation option. Further studies are needed to confirm this finding, elucidating the underlying molecular mechanisms of quercetin.

## 1. Introduction

Telomeres are nucleoprotein complexes capping the 23 pairs of linear chromosomes of each human cell. They contain tandem repeats of non-coding DNA motifs, specifically, in vertebrates, 5′-TTAGGG-3′ repeat units and their interacting protein partners, including a six-protein telomere-binding protective shelterin complex and a ribonucleoprotein (RNP), telomerase [1,2,3,4]. Importantly, the shelterin complex shields chromosomal ends of eukaryotic cells from recognition as double-stranded breaks (DSBs) [3]. The potential identification of telomeres as exposed DNA damage sites can lead to telomere fusion, degradation, recombination, or even telomere shortening (TS) [5], thereby compromising genomic stability [6].

More specifically, cellular proliferative rounds cause TS because of the end replication problem [7]. When telomeres reach a critically short length, they lose the protection of the shelterin protein complex, compromising their function. This leads to the triggering of the DNA damage response (DDR), inducing the activation of p53 transcription factor and the subsequent upregulation of cell-cycle inhibitors p16Ink4a and p21Cip1, resulting in cellular senescence, followed by apoptosis [8].

The most pronounced characteristic of senescent cells is their growth-arrested condition. In parallel, senescent cells are characterized by increased secretion of growth factors, cytokines, chemokines, proteases, and lipids, forming the senescence-associated secretory phenotype (SASP), thus sustaining low-grade inflammation [9]. On the other hand, uncapped telomeres can lead to numerous chromosome abnormalities and end-to-end fusion of chromosomes due to the absence of cell-cycle checkpoint mechanisms, resulting in genomic instability and widespread cell death [8].

Whereas TS is linked to aging and senescence, senescence does not always indicate cell aging [10]. Accordingly, a growing body of evidence proposes that the progression of age-related disorders may depend on the rate of TS [11,12,13,14,15,16]. Significantly, research has shown that telomere length (TL)—short, critically short, and critically long—is a recognized marker of biological aging and is considered a putative indicator of the cellular wellness of a person, with some predictive value for the risk of age-related illnesses like diabetes, osteoporosis, autoimmune diseases, neurodegenerative diseases, cardiovascular diseases, and some types of cancer [11,12,13,14,15,16,17]. For instance, TL has demonstrated utility in identifying patients with type 2 diabetes mellitus (T2DM) at risk of mortality [18]. The definitive role of TL in age-related diseases (ARDs) is still debated.

Relevant to this context, T2DM constitutes a global epidemic characterized by chronic dysglycemia, intertwined with heightened oxidative stress and inflammation [19], as elaborated elsewhere [20], a combination strongly correlated with accelerated TS. These interconnected processes may further contribute to genomic instability [21] and an increased vulnerability to vascular complications [22,23]. The interwoven relationship between telomere uncapping, senescence burden [23], and T2DM etiology underlines the necessity for innovative therapeutic interventions to preserve telomere integrity and mitigate disease progression in this population group.

Proceeding from this premise, emerging research suggests potential in targeting senescent cells and telomeres for the treatment and prevention of ARDs, and particularly T2DM [24]. Both senolytic and senomorphic therapies, designed to either eliminate senescent cells or mitigate their adverse effects, are being explored as possibly favorable interventions for age-associated morbidities [24,25].

The evolving field of telomere-targeted therapies, including senolytics, senomorphics, and potential telomerase activators, represents a promising research frontier in the pursuit of longevity and healthspan [26,27]. Nevertheless, telomerase activators have been implicated in oncogenesis concerns [27,28,29], hinting at a prerequisite for rigorous research and evaluation of the safety and regulatory efficacy of these interventions before their therapeutic potential in the context of healthy aging and age-related disease management can be fully understood.

The implications of telomere dysfunction and cellular senescence possibly associated with ARDs have instigated investigations into potential senotherapeutic interventions [8,30]. Quercetin, a flavonoid found ubiquitously in plant-derived dietary sources [31], has attracted considerable research interest as a potential therapeutic agent for mitigating cellular senescence [32,33,34]. This polyphenolic compound with potent antioxidant and anti-inflammatory properties [35,36,37] may exert a dual, pleiotropic action as both a senolytic and senomorphic [32,33,34]. The former indicates it can function by selectively inducing apoptosis of senescent cells and the latter implies the potential ability of modulating, particularly attenuating the SASP, possibly rendering it a promising candidate for mitigating the adverse consequences of cellular senescence and telomere dysfunction and potentially paving the way for innovative phytotherapeutic interventions in the supportive care management of ARDs.

The complex interplay between telomere dysfunction and T2DM pathophysiology generates mixed methodology hypotheses, justifying a combined laboratory and clinical approach to synchronically examine the variation of telomere status and disease progression in this vulnerable population group. Therefore, leveraging our prior work elucidating the safety and clinical efficacy aspects of quercetin nutritherapy [20,38], revealing a presumed improvement in standard risk factors, this randomized controlled before-and-after trial further delves into the potential of quercetin to modulate whole blood absolute TL in patients with T2DM, providing exploratory insights and putatively representing a novel nutritional senotherapeutic piece of knowledge to ARDs.

## 2. Results

**Figure 1:**
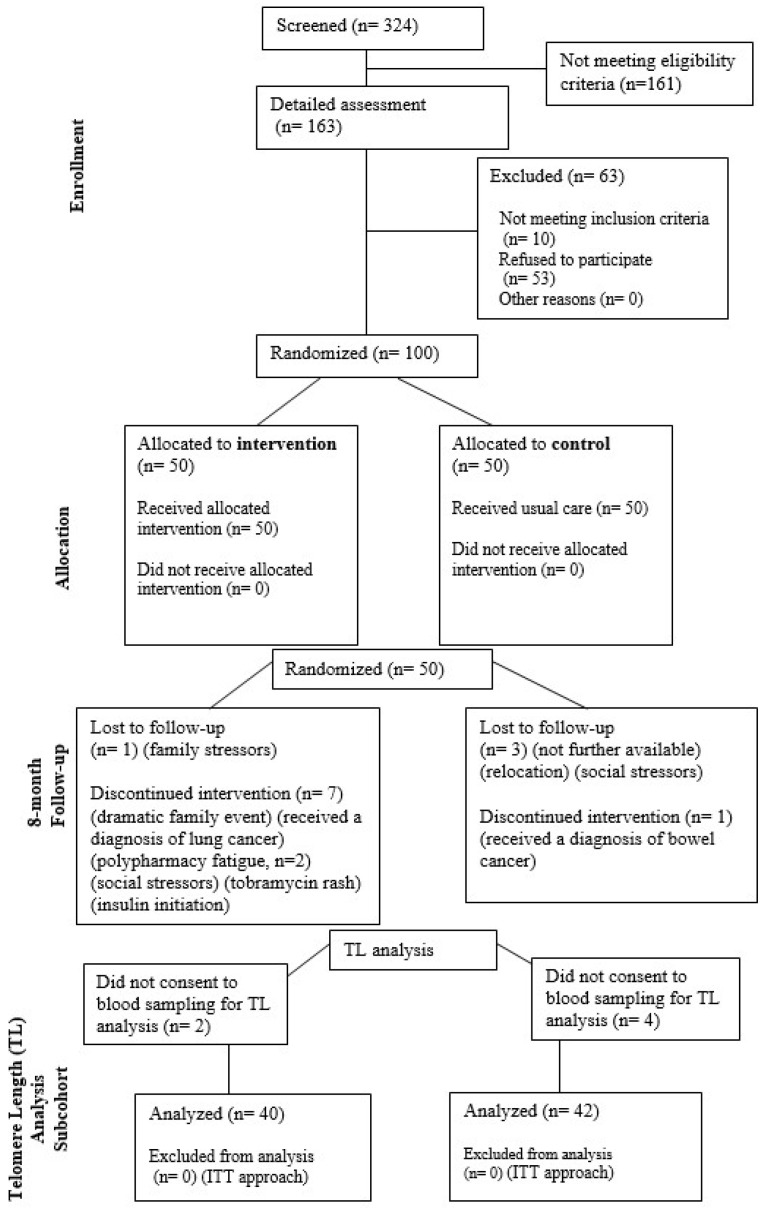
Flow diagram for the TL analysis subcohort, aligning with CONSORT standards.

Of the 88 patients concluding the trial [20], 82 voluntarily consented to blood sampling for absolute whole blood TL measurements by quantitative polymerase chain reaction (qPCR), as shown in the report-specific flowchart (Figure 1). This subsequent analysis was therefore confined to this subcohort of 42 control and 40 intervention participants. Aligning with the study design, adherence to the nutraceutical allocation was consistent within the treatment group. 

The baseline characteristics of the 82 randomized patients with T2DM consenting to blood sampling for whole-blood TL measurements are displayed in Table 1 using chi-squared and Student’s *t*-tests for comparison. The mean age of participants was 66.3 ± 7.4 for the intervention and 67.8 ± 6.2 years for the usual care group. The groups were well-balanced in sex, age, and nationality attributes (*p* > 0.05). Both groups were under treatment with oral antidiabetic pills (viz. metformin, SGLT-2 and DPP-4 inhibitors, sulfonylurea, or their combinations, *p* > 0.05), while eight in each group were additionally treated with GLP-1 agonists (*p* = 0.913). A balanced distribution of lifestyle risk factors between both groups was noted, without statistically significant intergroup differences in their prevalence (smoking, alcohol consumption, high body weight, physical inactivity, and lack of fruit consumption; *p* > 0.05). Also, no significant differences were discerned between the groups regarding the prevalence of multimorbidity, polypharmacy, or the mean duration since T2DM diagnosis (*p* > 0.05). Specifically, the occurrence of the most common age-related conditions (hypertension, dyslipidemia, chronic inflammatory airway diseases; CIADs, depression and anxiety disorder) was similar (*p* > 0.05) between the study groups of this subcohort.

Overall, both groups were comparable at baseline, as demonstrated in Table 1 and Table 2. The inter- and intra- group comparisons of health indicators of the 82 patients with T2DM randomized to intervention and control groups included in this analysis, from the starting point to the endpoint (8 months) of the study are presented in Table 2. The intervention group demonstrated a statistically significant improvement in night-time sleep duration, while the control group experienced a decrease (0.8 vs. −0.5 h, *p* < 0.001). Health self-assessment scores significantly improved in the intervention. In contrast, the control group experienced a decrease, yielding a statistically significant intergroup difference (1.5 vs. −0.6, *p* < 0.001). Between the two groups, no significant changes were observed in body mass index (BMI) values (*p* > 0.05). Systolic blood pressure significantly decreased in the intervention group, while it increased in the control group (−6.3 vs. 0.5 mmHg, *p* = 0.02). Neither inter- nor intra-group comparisons revealed significant alterations in diastolic blood pressure (*p* > 0.05). Between-group analyses showed no significant changes in total cholesterol, whereas the cholesterol ratio insignificantly decreased in the intervention group (*p* = 0.07). A statistically significant decrease in the glycated hemoglobin (HbA1c) measures of the intervention group was observed compared to the control group (−0.28 vs. 0.03%, *p* = 0.008). Average TL per chromosome end significantly increased in the intervention group (from 5.11 ± 1.35 to 5.63 ± 2.08 kb), while the control group experienced a decrease (5.19 ± 1.76 to 4.88 ± 1.19 kb; 0.52 vs. −0.31 kb, *p* = 0.048) over the eight-month supplementation period.

**Figure 2:**
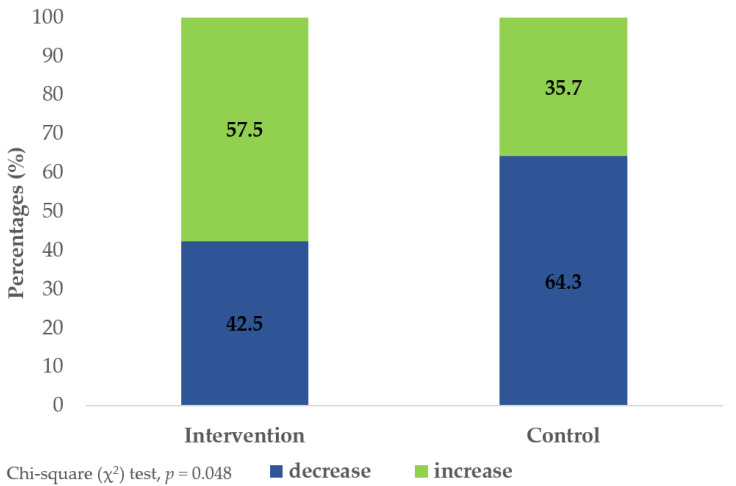
Frequency of telomere length (TL) changes (increase or decrease) in patients with type 2 diabetes mellitus (T2DM) consenting to blood sampling for whole-blood TL measurements, randomized to intervention (n = 40) and control (n = 42) groups over the 8-month study period.

The percentage frequencies of TL changes, as increase or decrease, from baseline to the endpoint at 8 months for the 82 consenting patients analyzed in this report are illustrated in Figure 2. A significant change in TL dynamics was observed between the two groups as the intervention group exhibited a higher frequency of TL increase compared to counterparts (57.5% vs. 35.7%) (*p* = 0.048).

The results of multiple logistic regression analyses examining the prognostic factors associated with an increase in average TL (versus decrease) over the 8-month subcohort-study period are delineated in Table 3. Except for the crude model, three more models were constructed, each incorporating a progressively comprehensive set of covariates. In the unadjusted model (crude), the intervention group exhibited a 2.44-fold higher odds of TL increase compared to the control group (*p* = 0.05). After adjusting for baseline TL through model 1, the intervention group maintained a 2.87-fold increased odds of TL increase (*p* = 0.036). Even after further accounting for sex and age attributes as additional confounders, the association was consistently statistically significant (model 2, odds ratio; OR = 2.90, *p* = 0.04). When the model included an expanded range of confounders, i.e., lifestyle risk factors, multimorbidity, polypharmacy, and years since T2DM diagnosis, an amplified effect was demonstrated (OR = 3.48, *p* = 0.026) (model 3). Furthermore, higher baseline TL was consistently related with a significantly decreased likelihood of telomere increase in patients with T2DM after controlling for multiple covariates (OR < 1.00, *p* < 0.01).

## 3. Discussion

The results deriving from this subcohort are in parallel with our prior clinical findings [20,38], further solidifying the promising potential of quercetin as an adjunct nutritional senotherapeutic intervention for T2DM. They also add a compelling layer of evidence, expanding previous knowledge on the overall health status, underscoring its role in disease progression and compelling further investigation of the translational senolytic potential of quercetin on diabetic sequalae. As a matter of fact, to the best of our knowledge, our study constitutes the first randomized controlled before-and-after trial to assess the impact of quercetin on absolute TL of an age-related disease while concurrently examining clinical features in primary care attendees.

Only one randomized controlled trial (RCT) on seniors with metabolic syndrome has evaluated the influence of quercetin supplementation on leukocyte TL (LTL) but found no changes post-supplementation [39]. Nevertheless, the limited 3-month study period, as well as the lower daily dosage of 240 mg, might have constrained the imprint of this intervention. Conversely, in our study, the intervention group received quercetin intermittently: for 12 weeks, followed by an 8-week washout period and another 12 weeks of supplementation. Another point for discussion within the limitations of the mentioned study includes its measurement of TL, which was based on a relative TL evaluation (T/S ratio) [40,41,42,43,44].

This analysis provides initial findings suggesting that quercetin could offer advantageous nutriprotective benefits in a population with T2DM. Specifically, within this subcohort of 82 patients who underwent TL blood sampling, significant positive effects were noted on TL and clinically relevant improvements, including nocturnal sleep duration and subjective health status. There was also a decrease in systolic blood pressure and glycated hemoglobin and a marginally insignificant decrease in the cholesterol ratio. BMI, diastolic blood pressure, and total cholesterol remained largely unchanged.

As the potential molecular mechanisms underlying these protective effects have been extensively explained in our prior publications [20,38] and the pre-defined primary endpoint of this study was the impact on TL [45], the current analysis will not reiterate previously explored mechanistic aspects. It will instead focus on a proof-of-concept approach.

Our results regarding the absolute TL of whole blood samples are consistent with previous research in the general population of similar age range [43,46,47,48,49]. Notably, our results lean towards the lower end of the observed TL distribution of healthy populations [50,51], aligning with the previous literature on the association between shorter telomeres and diabetes [52,53,54,55]. The observed increase in the absolute TL measurements within the intervention group, independent of potential confounders, suggests a potential reduction in the proportion of the senescence-inducing telomeres, potentially conferring beneficial effects on cellular health and longevity. While our methodology provides a measure of telomere length, it is also possible that the removal of cells with shorter telomeres could contribute to the observed net increase. Therefore, the detected increase in TL can only be discussed as a preliminary observation.

It is also important to highlight that when measuring TL in whole-blood samples, the absolute TL measurement primarily reflects the TL of the most abundant cells in blood, which are circulating immune cells, while other cell types present in blood typically do not contribute significantly to the overall measurement. The senolytic effects of quercetin have been investigated in endothelial and adipose tissue cells [56,57] to date, yet the evidence is less well-established regarding the circulating immune cells. Our exploratory results underline the necessity for further biological studies in this nascent field.

The existing literature on the consequences of different nutritive interventions on TL reveals divergent findings, with scant evidence from RCTs [58] demonstrating both beneficial and nuanced effects. Notably, the D-Health Trial, involving regular monthly vitamin D supplementation, did not provide a protective effect on telomere attrition in older individuals already possessing sufficient levels of vitamin D [59]. Similarly, in a subcohort of the double-blind, n-3 PUFA RCT of 106 subjects over 4 months, no significant differences in TL were observed between the intervention and control groups [60]. Conversely, a 6-month RCT including 33 adults over 65 years old with minor neurocognitive deficit suggested that omega-3 fatty acid intake may attenuate TS [61]. A two-year-long RCT with 162 participants examining LTL after walnut consumption indicated a potential trend towards LTL preservation in older adults, possibly associated with its alpha-linolenic acid and polyphenol-rich content [62]. Furthermore, a smaller, one year-long study with 65 individuals provided preliminary evidence of correlation between the supplementation with vitamins of the B-complex and the mitigation of telomere attrition [63].

Our findings add to the limited body of research on senescence-targeting interventions exploring telomere biology in humans [64]. Focusing on the available senolytic interventions, a prospective study investigated the use of hyperbaric oxygen therapy (HBOT) in 35 healthy adults over 64 years old. This study employed flow-fluorescence in situ hybridization (Flow-FISH) analysis of peripheral blood mononuclear cells (PBMCs) and reported a significant increase in TL across immune cell subsets, specifically, T helper cells, cytotoxic T cells, natural killer cells, and B cells, with the latter exhibiting a more pronounced effect [65]. In contrast, a longitudinal study evaluated the combination of dasatinib and quercetin (D + Q) for six months, followed by an identical six-month regimen plus fisetin one year later, in 19 participants utilizing a DNA methylation (DNAm)-based clock. Distinct effects on TL were revealed. The initial regimen resulted in a decrease in TL at the 3- and 6-month time points, whereas the subsequent addition of fisetin appeared to mitigate TS after one year of treatment [64].

An intriguing perspective unveiled from this analysis urges to be elucidated: the interconnection between favorable clinical and cellular homeostasis aspects following quercetin supplementation. These comprised ameliorated nocturnal sleep duration, well-being perception, blood pressure normalization and improved glycemic regulation. Interestingly, TS has been associated with poor sleep patterns [66,67,68], compromised psychophysiological health [69,70,71], hypertension [72,73], and impaired glycemic control [54,74].

The observed positive effects of quercetin on TL could be attributed to various, interrelated mechanistic reasons with yet unresolved intricacies and primarily to its capacity for mitigating oxidative stress and inflammation [35,36,37], both of which are known to accelerate TS [75,76]. This antioxidant protection potentially enables enhanced telomerase stability [3,77]. Furthermore, building upon the previously discussed contextualized rationale regarding its potential senolytic and senomorphic properties [32,33,34], quercetin may exert a preventive effect on TS by reducing the burden of senescent cells. Additionally, research suggests that quercetin could act as a regulator of key signaling pathways crucial for preserving cellular homeostasis, modulating sirtuins (especially activating SIRT1 and potentially dose-proportionally regulating SIRT6 [78,79,80,81]) and the AMP-activated protein kinase (AMPK) enzyme [82,83,84].

Remarkably, quercetin might exert intricate effects on telomerase activity, suggesting a potential for differential modulation in diverse cellular milieux and dose-dependent contexts, as bolstered by its polyphenolic property [85]. Although the relevant research is rapidly evolving, highlighting the necessity for further biological studies, the current literature proposes that in cancer cells, it inhibits telomerase activity [86,87,88], consistent with findings for other flavonoids [85,88], while concurrently selectively modulating the expression of POT1, TRF1, and TRF2 proteins [89]. This inhibitory effect in cancer cells, combined with an observed activation of hTERT in certain cell lines [90], raises the intriguing possibility that it may preferentially regulate telomerase activity in healthy human cells [91], potentially favoring the activation of hTERT or the expression of telomere-protective proteins and subsequent telomere elongation. Whether this proposed selective telomerase activation hypothesis is valid and translates to tangible benefits in telomeric elongation could initiate a pathway for ongoing investigation. Additionally, the literature supports its influence on epigenetic modifications, viz. DNA methylation, histone acetylation, and microRNA regulation [91,92], hinting at a broader role in regulating cellular processes involved in telomere maintenance.

### Study Strengths and Limitations

A key strength of our study lies in its comprehensive assessment of telomere dynamics in T2DM, employing accurate, high-throughput absolute TL measurements by qPCR [40,41,42,43,44]. Considering our RCT prioritized the metabolic and clinical implications in a substantial population with T2DM, we opted for the more practical qPCR measurements through commercial kits, which are informative at the cell-population level. Even though qPCR provides an individual’s average telomere data based on the specific amplification of telomere repeats, it is not considered a precise method [40]. The primary limitation of qPCR is its incapacity to offer details regarding the TL variations among cells and chromosomes [40]. For this reason, quantitative fluorescence in situ hybridization (qFISH) is the preferred technique for high-resolution evaluation of TL of cells in each chromosome at the single-cell level [93,94]. In addition, qFISH effectively captures even the extremely short telomeres [95], unveiling more precise TL alterations at the single-cell level [96,97] and could provide a more nuanced explanation of the observed effect on TL. While our study involved an open-label, non-placebo-controlled design, the primary outcome, TL, was directly measured using objective qPCR assays. Likewise, the staff conducting hematology tests for secondary outcomes were blinded to group allocations, further minimizing the risk of measurement bias. Furthermore, this study was prospectively registered within the International Standard Randomized Controlled Trial Number (ISRCTN) registry [45]. This work is the content of an ongoing PhD thesis, with no external funding, with permissions from all involved institutional bodies, after full protocol submission, and later performed in alignment with its protocol design requirements. Homogenous procedures across all study groups enabled for a standardized evaluation of the ascertained outcomes. Additionally, an intention-to-treat (ITT) approach was employed, which attenuates the potential for overestimation of intervention efficacy [98].

By incorporating absolute TL measurements from whole blood alongside relevant clinical biomarkers, we have attempted a deeper, integrative understanding of the intricate relationship between aging processes and T2DM. This approach provides unique, multilateral insights into how senotherapy may palpably influence telomere dynamics and interact with other cardiometabolic health parameters, potentially targeting disease progression and improving outcomes in patients with T2DM. Also, the 8-month rigorous RCT design, despite the availability of a subcohort of samples, equipped us with a power of detection of at least 80%, which should be mentioned. Moreover, the models fitted in the multiple logistic regression analysis were adjusted for several confounders. Another limitation is that we could not assess further biological variables which might have been proven helpful to explain the underlying mechanisms. Given the emerging role of cellular senescence in both the development of diabetes [99] and its complications [23], our study offers potentially relevant insights into promising therapeutic avenues for this disease. Nonetheless, our study design was not specifically tailored to address these elements. Future studies should include more extensive, larger-scale, blinded, placebo-controlled, dose–response assessment designs examining disease progression and complications so as to verify the senolytic results and to enable further decipherments.

## 4. Materials and Methods

### 4.1. Study Design, Participants, Phytonutrient

We conducted a prospective, randomized controlled before-and-after trial within a primary healthcare (PHC) facility in Heraklion, Crete, in the context of a PhD thesis of the Clinic of Social and Family Medicine, Department of Social Medicine, School of Medicine, University of Crete, with the aim to evaluate the clinical utility and potential of quercetin supplementation as an adjunct support for patients managing T2DM. Data were collected from registered patients within the 4th Local Health Unit of Heraklion (TOMY). Participants over 50 years old with an established diagnosis of T2DM and treated with non-insulin medications were enrolled in the trial between February and May 2023 after providing informed consent, as described in our previous report [20].

Of the 324 patients initially identified as candidates, 100 were ultimately randomized into two parallel groups after stratified randomization (1:1) ensuring balanced age and sex distribution: a control group (CTR, n = 50) receiving standard care and an intervention group (INT, n = 50) receiving quercetin dihydrate (500 mg/day) supplementation for two 12-week periods interspersed with an 8-week washout. Specifically, the INT received quercetin as an adjunctive treatment to their existing regimen and the control group did not, while both groups received uniform guidance. Further details on the phytonutrient supplement specifications and strategies to enhance participants’ adherence to protocol requirements were formerly reported [20,38]. Participant flow throughout the randomized trial has also been delineated elsewhere [20], outlining reasons for attrition. Scheduled follow-up assessments were conducted in person: at baseline, at 3 months for a randomized subset, and at study endpoint. Participants underwent comprehensive health evaluations, and standardized fasting blood samples were collected at baseline and study conclusion, as mentioned in our previous work [20,38]. Participants self-rated their overall health status on a scale of 0 to 10, with 0 indicating extremely poor and 10 reflecting optimal health, for the needs of the health-self-assessment variable. TL was identified as the primary outcome of this study, as detailed in the study registry [45]. Of the 88 participants reaching the final study endpoint at 8 months, 82 consented to venipuncture for collection of blood samples in ethylenediaminetetraacetic acid (EDTA)-coated tubes.

Following gentle inversion to ensure thorough mixing, samples were securely stored at −20 °C, under strict quality and tracing control mandates, until genomic DNA extraction. Genomic DNA was subsequently isolated from the whole blood of these consenting patients. qPCR quantified absolute TL, and the resulting data are outlined in the current report.

#### Sample Size Estimation

Sample size estimates were determined at the clinical design stage as 45 participants per group using G*Power 3.1.9.7 [100,101], treating TL as a continuous variable, in consistency with calculations in methodologically comparable studies [60,102,103,104,105,106], and exceeding that of similar studies [107,108,109]. A post-hoc power analysis was conducted on the available sample, yielding a statistical power of at least 80%.

### 4.2. Telomere Length Evaluation

TL assessments were conducted at the Department of Morphology, Laboratory of Toxicology, School of Medicine, University of Crete, which has established expertise in this research field [110,111] under consistent experimental conditions.

#### 4.2.1. DNA Extraction

Genomic DNA was extracted from human whole-blood samples using the NucleoSpin^®^ Blood QuickPure kit (Macherey-Nagel, Düren, DE (Germany), catalog #740569.250, LOT no: 2311-003, exp.: 2026-02), as per manufacturer’s recommendations. All samples were lysed with proteinase K and lysis buffer at 70 °C and to ensure optimal DNA yield, an extended lysis protocol was implemented, involving further incubation for 30 min with intermittent vortexing. Following lysis, DNA was bound to silica columns, washed, and eluted with pre-warmed elution buffer, according to the kit’s instructions. DNA purity and integrity were evaluated utilizing a NanoDrop ND-1000 spectrophotometer (Thermo Fisher Scientific, Wilmington, DE, USA) and accompanying NanoDrop ND-1000 v.3.3 software (Coleman Technologies Inc., Langley, BC, Canada). Successfully extracted samples were then diluted to a working concentration of 5 ng/μL for downstream analysis and subsequently short-term stored at 4 °C.

#### 4.2.2. Absolute Quantification of Telomere Length

qPCR quantified the absolute TL. Specifically, the Absolute Human Telomere Length Quantification qPCR Assay Kit (ScienCell, Carlsbad, CA, USA, Cat #8918) was used to quantify the average length of telomeric ends on each chromosome of pure genomic DNA (gDNA), isolated from human blood samples, as described previously. qPCR was performed on a Stratagene Mx3005P qPCR thermocycler (Agilent Technologies Inc., Santa Clara, CA, USA) through MxPro v.4.1.0.0 software. Following the manufacturer’s protocol, two qPCR reactions were performed for each gDNA sample: one with telomere-specific (TEL) and one with single-copy reference (SCR) primers, targeting a 100 base-pair region on chromosome 17 for internal normalization. A reference gDNA sample of known TL (REF) provided in the kit served as a standard. Duplicate qPCR reactions were performed for each gDNA sample (4 qPCR reactions in total) on the same 96-well-plate, consistent with prior methodology [104,112], using water as the negative control and reference gDNA (REF) as the positive control in each run. In more detail, reactions contained 5 nanograms of gDNA template, combined with 2X GoldNStart TaqGreen qPCR Master Mix and either TEL or SCR primers. Samples from the intervention and control groups were run simultaneously on the same well plate. Additionally, minimal variation in Cq values was tolerated for all samples, while the entire experimental analysis was repeated in independent assays to ensure reliability and performance reproducibility [105,113,114,115]. The qPCR thermal cycling parameters consisted of a ten-minute preliminary denaturation at 95 °C, followed by 32 cycles of denaturation at 95 °C (20 s), a 20 s annealing at 52 °C, and a 45 s extension at 72 °C. Relative differences in TLs were calculated using the Livak comparative (ΔΔCq) method [116,117] through tabulation. Briefly, the difference in average quantification cycle (Cq) values between the target and reference samples was calculated for both telomere (TEL) and single-copy reference (SCR) amplicons (ΔCq TEL and ΔCq SCR). The ΔΔCq value was then obtained by subtracting ΔCq SCR from ΔCq TEL. The fold change in TL relative to the reference was calculated as 2^−ΔΔCq^. Absolute TL per diploid genome was determined by multiplying the relative TL by the known TL of the reference gDNA sample. Average TL (in kb) per chromosome end was calculated by dividing the total TL per diploid genome by 92. Amplification plots derived from standard curves assessed the efficiency and specificity of the qPCR reactions.

### 4.3. Statistical Analysis

Statistical analyses were conducted using the SPSS software (IBM SPSS Statistics for Windows, Version 25.0. Armonk, NY, USA: IBM Corp). Data normality was assessed with Blom’s method (Q-Q plot), and χ^2^ and Student’s *t*-test methods were implemented, respectively comparing categorical and continuous data between the two groups. Most of the results were presented with measures of location and dispersion or frequency distributions. Four multiple logistic regression models were constructed to assess the association between quercetin intake and the likelihood of TL increase, considering potential confounding factors, including established lifestyle-habit risk factors, previously associated with health outcomes [118,119,120]. The improvement in explained variance for TL changes was demonstrated by increasing Nagelkerke R^2^ determination values across sequential models, indicating a progressively enhanced model fit [121].

### 4.4. Ethical Compliance and Approvals

This RCT was registered with ISRCTN [45] and received ethical approval from the University of Crete Research Ethics Committee (REC-UoC; 104/20-08-2021) and the 7th Health District of Crete, Heraklion (6380-14/02/2022), while aligning with international ethical and regulatory standards [122,123,124,125,126,127]. This RCT also adhered to the International Council for Harmonization (ICH) E18 guideline [128] on genomic sampling and data management, adopted by the European Medicines Agency [129].

## 5. Conclusions

Our study provides valuable insights through unifying hypotheses and integrating clinical parameters and telomere senescence pathways. We offer a deeper understanding of the relationship between aging processes and T2DM to assess the impact of quercetin intake in patients with T2DM. In this context, we highlight the potential of incorporating quercetin into PHC supportive interventions for T2DM. We report that quercetin, as a complementary phytonutritive senotherapy alongside standard pharmacological regimens, might propose an integrative care plan, as evidenced by its emerging potential to significantly increase the average telomere length of the intervention group, along with improvement of sleep, well-being perception, blood pressure and glycemic regulation. These findings create possibilities for targeting disease progression and improving patient outcomes, although future larger-scale, multi-center, placebo-controlled, dose–response assessment and blinded studies are needed for validation. Importantly, our work indirectly showcases how lifestyle choices might affect the progression of T2DM and underscores the value of implementing TL measurements into clinical assessments for T2DM populations. This opens avenues enabling specific, informed research initiatives, and early identification of aberrant telomere shortening before and after overt clinical manifestations, potentially paving the way for ‘personalized medicine’ and multi-omics approaches to learn more about disease trajectory and monitoring.

## Figures and Tables

**Table 1:** Baseline descriptive characteristics of the 82 patients with type II diabetes (T2DM) consenting to blood sampling for telomere length (TL) measurements and randomized to intervention and control groups.

		Groups	
		Intervention (n = 40)	Control (n = 42)
		n (%)	
Sex	male	22 (55.0)	23 (54.8)
	female	18 (45.0)	19 (45.2)
Age, years	mean ± stand. dev.	66.3 ± 7.4	67.8 ± 6.2
Nationality	Greek	36 (90.0)	42 (100.0)
Lifestyle Risk Factors	smoking	13 (32.5)	15 (35.7)
	alcohol consumption	28 (70.0)	28 (66.7)
	high body weight	35 (87.5)	39 (92.9)
	physical inactivity	16 (40.0)	21 (50.0)
	not consuming fruits	2 (5.0)	2 (4.8)
	3+ factors	16 (40.0)	23 (54.8)
Multimorbidity	3+ chronic conditions	32 (80.0)	28 (66.7)
Polypharmacy	4+ medications	28 (70.0)	29 (69.0)
Years diagnosed with T2DM	mean ± stand. dev.	11.0 ± 7.1	10.3 ± 6.6

Chi-square (χ^2^) and Student’s *t*-tests: * *p* < 0.05.

**Table 2:** Levels of and changes in health indicators of the 82 patients with T2DM consenting to blood sampling for whole-blood TL measurements and randomized to intervention (n = 40) and control (n = 42) groups from the starting point to the endpoint of the study.

		Groups			
		Intervention	Control		Cohen’s *d* Effect Size
		Mean ± Stand. Dev.		*p*-Value	
Night-time sleep (hours)	beginning	6.2 ± 1.7	6.6 ± 1.5		
	8 months	7.0 ± 1.3	6.1 ± 1.7		
	***Δ*-change**	+0.8	−0.5	<0.001	0.93
	*p*-value	0.002	0.011		
Health self-assessment (scale 0 to 10, 10: excellent)	beginning	6.0 ± 2.1	5.9 ± 2.1		
	8 months	7.5 ± 1.7	5.3 ± 2.0		
	***Δ*-change**	+1.5	−0.6	<0.001	1.56
	*p*-value	<0.001	0.005		
Body mass index (kg/m^2^)	beginning	31.4 ± 5.1	30.6 ± 5.4		
	8 months	31.0 ± 5.1	30.3 ± 5.2		
	***Δ*-change**	−0.4	−0.3	0.780	0.05
	*p*-value	0.012	0.089		
Systolic blood pressure (mmHg)	beginning	130.7 ± 14.2	128.4 ± 13.8		
	8 months	124.4 ± 13.8	128.9 ± 16.5		
	***Δ*-change**	−6.3	+0.5	0.020	0.52
	*p*-value	0.003	0.804		
Diastolic blood pressure (mmHg)	beginning	76.0 ± 8.8	75.4 ± 10.7		
	8 months	77.4 ± 9.1	77.3 ± 8.8		
	***Δ*-change**	+1.4	+1.9	0.822	0.05
	*p*-value	0.276	0.196		
Total cholesterol (mg/dL)	beginning	154.9 ± 35.9	163.7 ± 37.6		
	8 months	160.4 ± 36.4	163.9 ± 44.4		
	***Δ*-change**	+5.5	+0.2	0.460	0.17
	*p*-value	0.122	0.966		
Glycosylated hemoglobin (HbA_1_c) (*%*)	beginning	7.03 ± 1.12	6.71 ± 0.79		
	8 months	6.75 ± 0.89	6.74 ± 0.94		
	***Δ*-change**	−0.28	+0.03	0.008	0.60
	*p*-value	0.005	0.655		
Cholesterol ratio	beginning	3.937 ± 1.962	3.567 ± 0.927		
	8 months	3.855 ± 1.403	4.109 ± 2.147		
	***Δ*-change**	−0.072	+0.752	0.070	0.41
	*p*-value	0.802	0.035		
Telomere length (kb)	beginning	5.11 ± 1.35	5.19 ± 1.76		
	8 months	5.63 ± 2.08	4.88 ± 1.19		
	***Δ*-change**	+0.52	−0.31	0.048	0.44
	*p*-value	0.120	0.235		

Student’s *t*-tests in *Δ*-changes (in bold) and paired samples *t*-tests between the baseline and study endpoint for each group (in grey).

**Table 3:** Multiple logistic regression analysis of TL increase (versus decrease) over the 8-month study period among the 82 patients with T2DM consenting to blood sampling for whole-blood TL measurements and randomized to intervention (n = 40) and control (n = 42) groups.

			Telomere Length Changes over the 8-Month Study Period (Increase vs. Decrease)		
Model	Prognostic Factors		Odds Ratio, OR	95% CIs	p-Value
Crude	Groups	control	1.00 (ref.)		
		intervention	2.44	1.00, 5.93	0.050
1	Baseline Telomeres (per unit change)		0.52	0.35, 0.79	0.002
	Groups	control	1.00 (ref.)		
		intervention	2.87	1.07, 7.70	0.036
2	Sex	male	1.00 (ref.)		
		female	1.64	0.60, 4.52	0.336
	Age (per year change)		0.95	0.88, 1.02	0.167
	Baseline Telomeres (per unit change)		0.49	0.32, 0.76	0.002
	Groups	control	1.00 (ref.)		
		intervention	2.90	1.05, 8.00	0.040
3	Sex	male	1.00 (ref.)		
		female	1.99	0.66, 5.96	0.221
	Age (per year change)		0.94	0.86, 1.02	0.137
	Baseline Telomeres (per unit change)		0.48	0.30, 0.76	0.002
	Lifestyle Risk Factors	0–2 factors	1.00 (ref.)		
		3+	2.23	0.75, 6.63	0.148
	Multimorbidity	0–2 chronic conditions	1.00 (ref.)		
		3+	0.61	0.17, 2.20	0.453
	Polypharmacy	0–3 medications	1.00 (ref.)		
		4+	0.93	0.28, 3.10	0.909
	Years Diagnosed with T2DM (per year change)		1.06	0.97, 1.15	0.186
	Groups	control	1.00 (ref.)		
		intervention	3.48	1.16, 10.41	0.026

R^2^ Nagelkerke estimations: Crude model R^2^ = 0.06; 1st model R^2^ = 0.25; 2nd model R^2^ = 0.30; 3rd model R^2^ = 0.33.

## Data Availability

The principal investigators retained exclusive access to the trial dataset, which was maintained under stringent security protocols on protected servers housed at the University of Crete. External researchers may be permitted to access the de-identified dataset contingent upon submission of a reasonable request and execution of appropriate data sharing agreements. To safeguard participant confidentiality and anonymity, all personally identifiable information is expunged prior to data dissemination. Trial Registration Number: ISRCTN13131584.
